# Supplementary material for: High risk of hypoxemic COVID-19 pneumonia in myasthenia gravis patients with type I IFN autoantibodies
Source: medRxiv. 2026 Apr 2:2026.03.27.26349525. Preprint. [Version 1] doi: 10.64898/2026.03.27.26349525 (PMC13060480; doi:10.64898/2026.03.27.26349525)
Supplement: Supplement 1 [file media-1.docx]

**Table 1: Baseline characteristics of the 86 MG patients sampled before the COVID-19 pandemic**

| Mean age (years) | 63 (range: 14-88) |
| --- | --- |
| Men (%) | 41 (48%) |
| Nature of MG-associated AAB (%)  Anti-AchR  Anti-MuSK  SN | 71.0  5.8  23.2 |
| Onset (%)  EOMG  LOMG | 23.3  76.7 |

**SN: seronegative**

**Table 2: Baseline clinical characteristics of the 85 MG patients infected with SARS-CoV-2**

| Mean age (years) | 54 (range: 23-90) |
| --- | --- |
| Men (%) | 41 (48%) |
| Nature of MG-associated AAB (%)  Anti-AchR  Anti-MuSK  SN  Unknown | 60 (70.6%)  3 (3.5%)  18 (21.2%)  4 (4.7%) |
| Type of MG (%)  Generalized  Ocular | 82 (96.4%)  3 (3.6%) |
| Onset (%)  EOMG  LOMG  Unknown | 43 (50%)  28 (33%)  14 (17%) |
| Treatment (%)  Immunomodulatory treatment  Corticosteroids  Azathioprine (AZA)  Mycophenolate mofetil (MFM)  Rituximab  Acetylcholinesterase inhibitors  Intravenous immunoglobulin  None | 64 (75%)  56 (66%)  30 (35%)  9 (11%)  9 (11%)  32 (38%)  10 (12%)  8 (9.5%) |
| Patients with thymoma (%) | 14 (17%) |
| Patients with thymectomy, without thymoma (%) | 20 (24%) |

**SN: seronegative**

**Table 3: Clinical manifestations of COVID-19 in the 85 MG patients infected with SARS-CoV-2**

| Men (%), mean age (years) | 41 (48%), mean age: 54 |
| --- | --- |
| Patients with hypoxemic COVID-19 (%), men (%), mean age (years)  Hospitalized (%)  ICU (%)  Oxygen-dependent  <6 L O_2_/min  >6 L O_2_/min  Unknown volume  Mortality (%)  Thymoma (%) | 48/85 (56%), men: 27 (58%), mean age: 57  47 (98%)  31 (65%)  48 (100%)  17 (35%)  28 (58%)  3 (7%)  8 (17%)  12 (25%) |
| Patients with non-hypoxemic COVID-19 (%), men (%), mean age (years)  Hospitalized (%)  ICU (for surveillance) (%)  Mortality (%)  Thymoma (%) | 37/85 (44%), men: 14 (38%), mean age: 50  9 (24%)  2 (5%)  0 (0%)  2 (5%) |

**Table 4: Summary of non-glycosylated type I IFN data for 85 MG patients infected with SARS-CoV-2**

| IFN(s) neutralized | Hypoxemic cases  *n*=48 (56%) | Non-hypoxemic cases  *n*=37 (44%) | Total  *n*=85 |
| --- | --- | --- | --- |
| IFN-α2 alone | 8 (17%) | 2 (5.4%) | 10 (12%) |
| IFN-ω alone | 0 (0%) | 1 (2.7%) | 1 (1.2%) |
| IFN-β alone | 0 (0%) | 0 (0%) | 0 (0%) |
| IFN-α2 and IFN-ω (+/- IFN-β) | 14 (29%) | 3 (8.1%) | 17 (20%) |
| IFN-α2 and IFN-ω and IFN-β | 1 (2.1%) | 0 (0%) | 1 (1.2%) |
| IFN-α2 and/or IFN-ω and/or IFN-β | 22 (46%) | 6 (16.2%) | 28 (33%) |

**Table 5: Summary of non-glycosylated type I IFN neutralization data for 85 MG patients infected with SARS-CoV-2 (100 pg/mL for IFN-α2 and IFN-ω, 10 ng/mL for IFN-β)**

| IFN(s) neutralized | Men  *N*=41 | Women  *N*=44 | <30 y.o.  *N*=10 | 30-70 y.o.  *N*=57 | >70 y.o.  *N*=18 | Total  *N*=85 |
| --- | --- | --- | --- | --- | --- | --- |
| IFN-α2 alone | 5 (12%) | 5 (11%) | 0 | 8 (14%) | 2 (11%) | 10 (12%) |
| IFN-ω alone | 0 | 1 (2.3%) | 0 | 1 (1.8%) | 0 | 1 (1.2%) |
| IFN-β alone | 0 | 0 | 0 | 0 | 0 | 0 |
| IFN-α2 and IFN-ω (+/- IFN-β) | 11 (27%) | 6 (14%) | 2 (20%) | 14 (25%) | 1 (5.6%) | 17 (20%) |
| IFN-α2 and IFN-ω and IFN-β | 1 (2.4%) | 0 | 0 | 1 (1.8%) | 0 | 1 (1.2%) |
| IFN-α2 and/or IFN-ω and/or IFN-β | 16 (39%) | 12 (27%) | 2 (20%) | 23 (40%) | 3 (17%) | 28 (33%) |

**Table 6: AAN-I-IFNs and COVID-19 severity according to the presence/absence of thymoma/thymectomy**

| Thymoma (*N*) | Yes: 14 | No: 71 | |
| --- | --- | --- | --- |
| Thymectomy (*N*) | Yes: 14 | Yes: 20 | No: 51 |
| Hypoxemic (*N*, %) | 12 (86%) | 6 (30%) | 30 (59%) |
| Non-hypoxemic (*N*, %) | 2 (14%) | 14 (70%) | 21 (41%) |
| AAB^+^ (*N*, %) | 9 (64%) | 4 (20%) | 15 (29%) |
| AAB^-^ (*N*, %) | 5 (36%) | 16 (80%) | 36 (71%) |
| Hypoxemic AAB^+^ (*N*) | 8 | 2 | 12 |
| Hypoxemic AAB^-^ (*N*) | 4 | 4 | 18 |
| Non-hypoxemic AAB^+^ (*N*) | 1 | 2 | 3 |
| Non-hypoxemic AAB^-^ (*N*) | 1 | 12 | 18 |
