# Supplementary material for: High risk of hypoxemic COVID-19 pneumonia in myasthenia gravis patients with type I IFN autoantibodies
Source: medRxiv. 2026 Apr 2:2026.03.27.26349525. Preprint. [Version 1] doi: 10.64898/2026.03.27.26349525 (PMC13060480; doi:10.64898/2026.03.27.26349525)
Supplement: Supplement 2 [file media-2.docx]

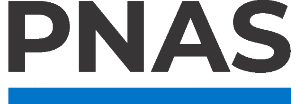


**Supplementary Information for**

High risk of hypoxemic COVID-19 pneumonia in myasthenia gravis patients with type I IFNs autoantibodies

Adrian Gervais, Astrid Marchal, Alexis Maillard, Tom Le Voyer, Jérémie Rosain, Quentin Philipot, Lucy Bizien, Jessica Peel, Axel Cederholm, Mélanie Migaud, Sylvie Pons, Kahina Saker, Pascal Laforet, Mélodie Aubart, Cyril Gitiaux, Catherine Biggs, Rafael Leon Lopez, Sarah Souvannanorath, Céline Tard, Aleksandra Nadaj Pakleza, Aude-Marie Grapperon, Nicholas Heming, Djillali Annane, Annie Verschueren, Shahram Attarian, Kévin Bigaut, Karolina Hankiewicz, Ludivine Kouton, Rocio-Nur Villar-Quiles, Cécile Cauquil, Marie-Céline Fleury, Emilie Rocher, Guillaume Nicolas, Eduardo de Paula Estephan, Maria da Penha Ananias Morita, Edmar Zanoteli, Zakaria Saied, Amine Rachdi, Amouri Rim, Samir Belal, Samia Ben Sassi, Annemarie Hübers, Emmanuel Faure, Isabelle Desguerre, Clémence Basse, Nicolas Girard, Vivien Béziat, Qiang Pan-Hammarström, Lennart Hammarström, Aaron Bodansky, Audrey V. Parent, Mark S. Anderson, Joseph L. DeRisi, , Sophie Demeret, Frédérique Truffault, Romain Fort, Florence Ader, Florent Wallet, Laurent Abel, Thierry Molina, Marie-Alexandra Alyanakian^&^, Rozen Le Panse^&^, Guilhem Solé^&^, Aurélie Cobat*, Nils Landegren*, Jean-Laurent Casanova*, Anne Puel*, Paul Bastard*^,@^, and Emmanuelle Jouanguy*

^&,^*Equal contributions

^@^ Correspondence: Paul Bastard ([paul.bastard@institutimagine.org](mailto:paul.bastard@institutimagine.org))

**This PDF file includes:**

Figures S1 to S4

**
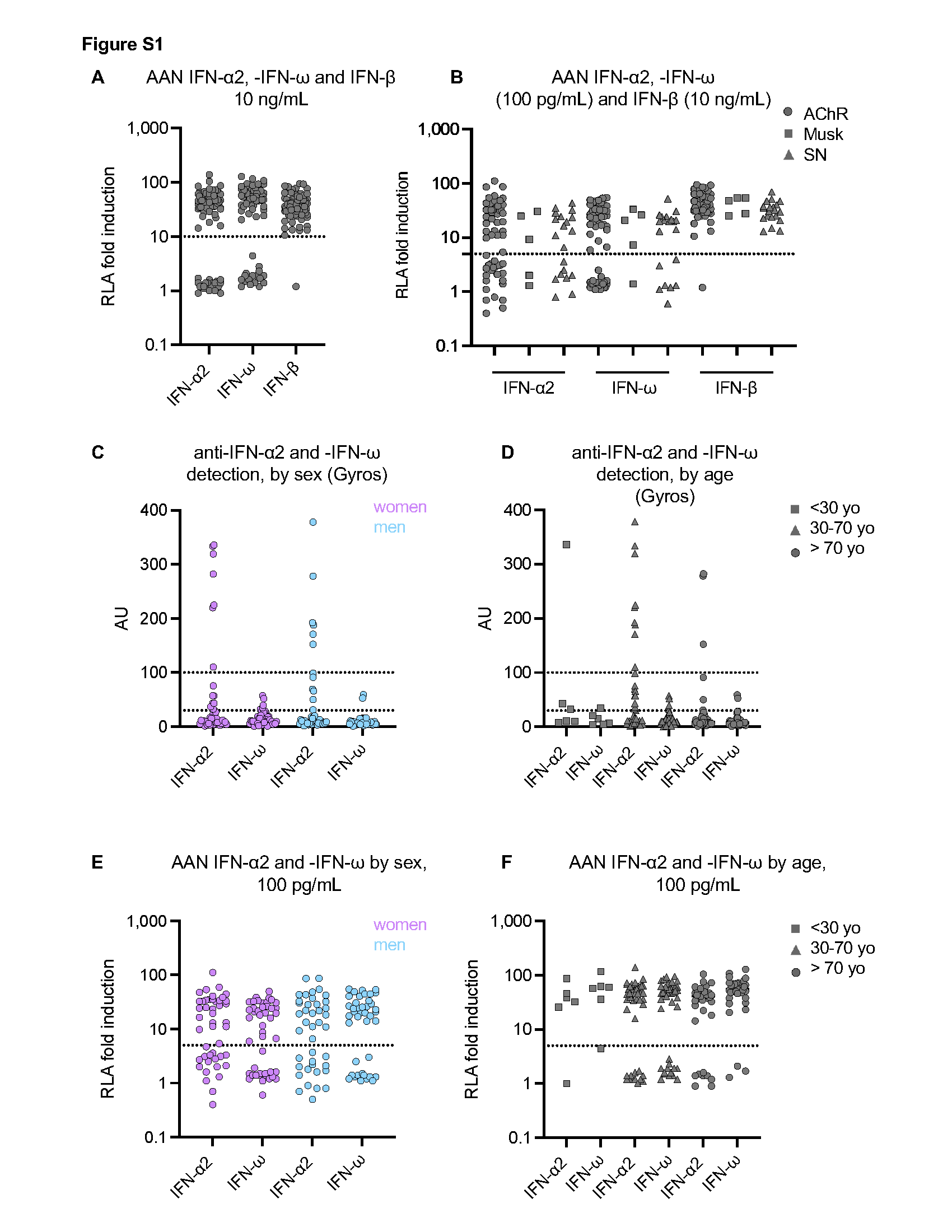
**

**Figure S1: Auto-Abs against type I IFNs in the 86 MG patients before the episode of COVID-19, by age, sex or MG serotype**

**(A)** AAN-I-IFNs neutralizing 10 ng/mL IFNs. **(B)** AAN-I-IFNs by MG serotype. **(C)** Detection by Gyros of auto-Abs against IFN-α2 and IFN-ω, by sex. **(D)** Detection by Gyros of auto-Abs against IFN-α2 and IFN-ω, by age group. **(E)** AAN-IFN-I, by sex. **(F)** AAN-IFN-I, by age group.


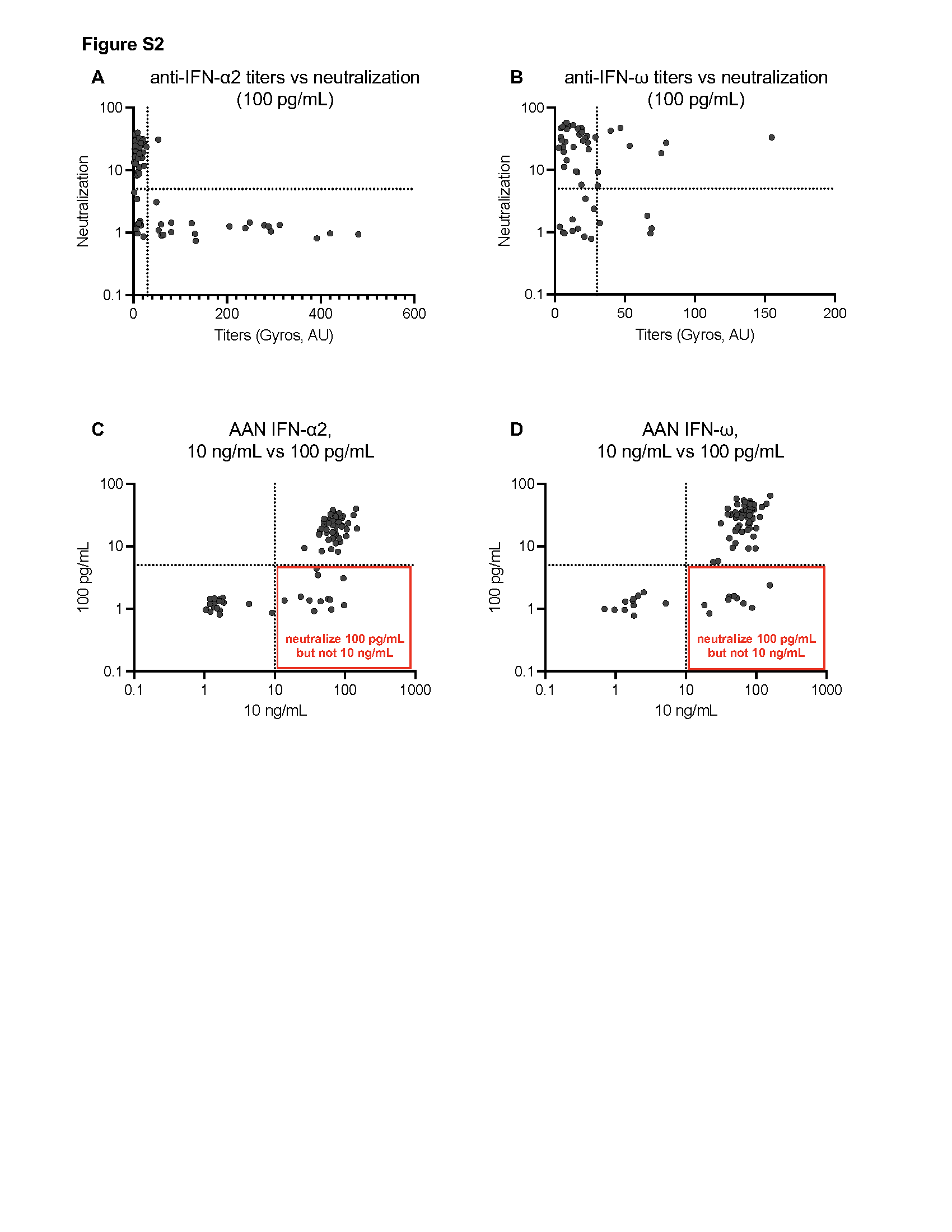


**Figure S2: Correlation of neutralization data with auto-Ab titers and correlation of neutralization between high and low IFN concentrations**

**(A)** Correlation between IFN-α2 titers determined by Gyros and neutralization of 100 pg/mL IFN as assessed by luciferase assay. **(B)** Correlation between IFN-ω titers determined by Gyros and neutralization of 100 pg/mL IFN as assessed by luciferase assay. **(C)** Correlation between the neutralization of IFN-α2 at concentrations of 100 pg/mL and 10 ng/mL, as assessed by luciferase assay. **(D)** Correlation between the neutralization of IFN-ω at concentrations of 100 pg/mL and 10 ng/mL, as assessed by luciferase assay.

**
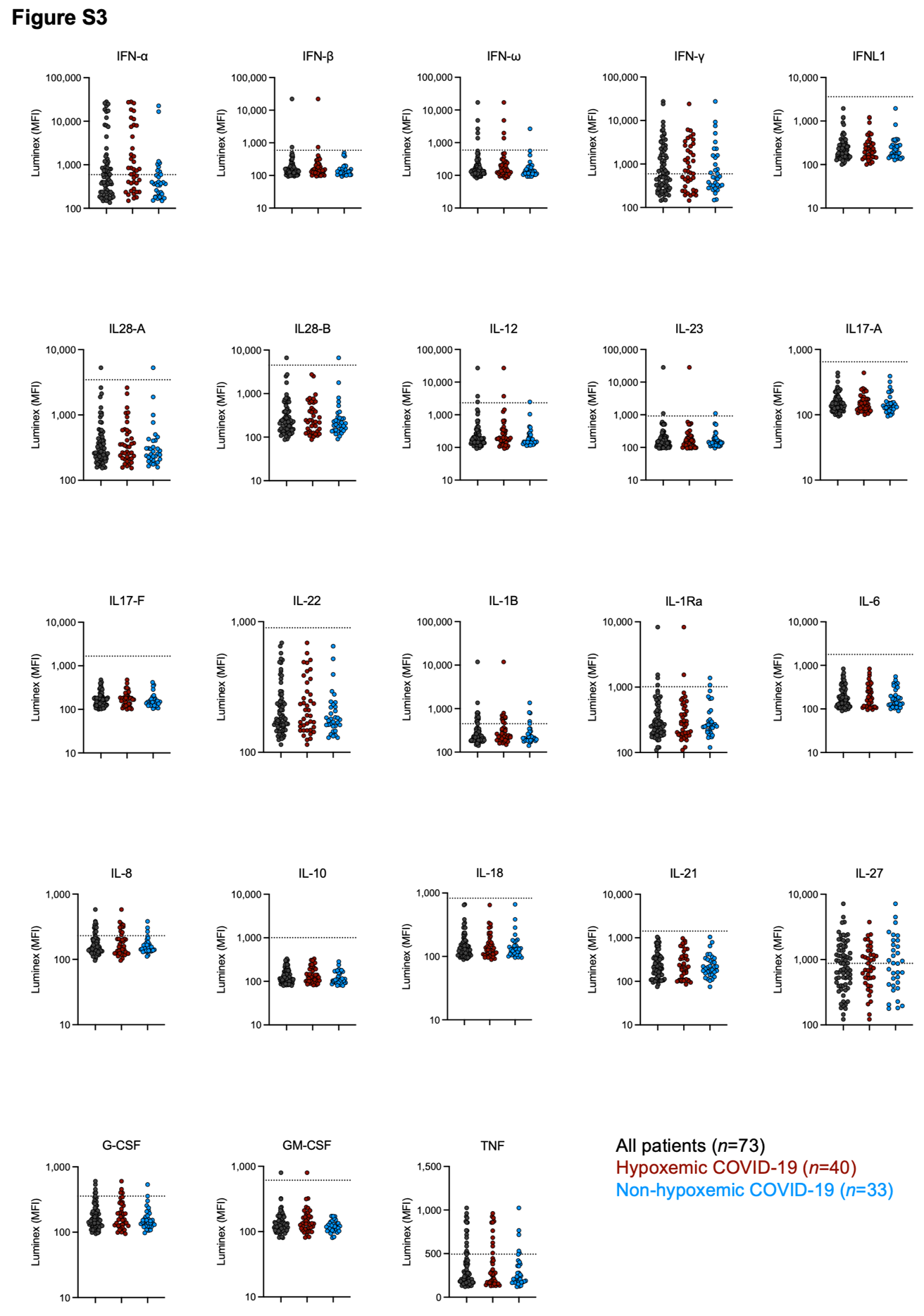
**

**Figure S3: Detection of a panel of auto-Abs against cytokines by Luminex® assays**

The threshold was defined as four standard deviations above the mean signal for healthy donors.


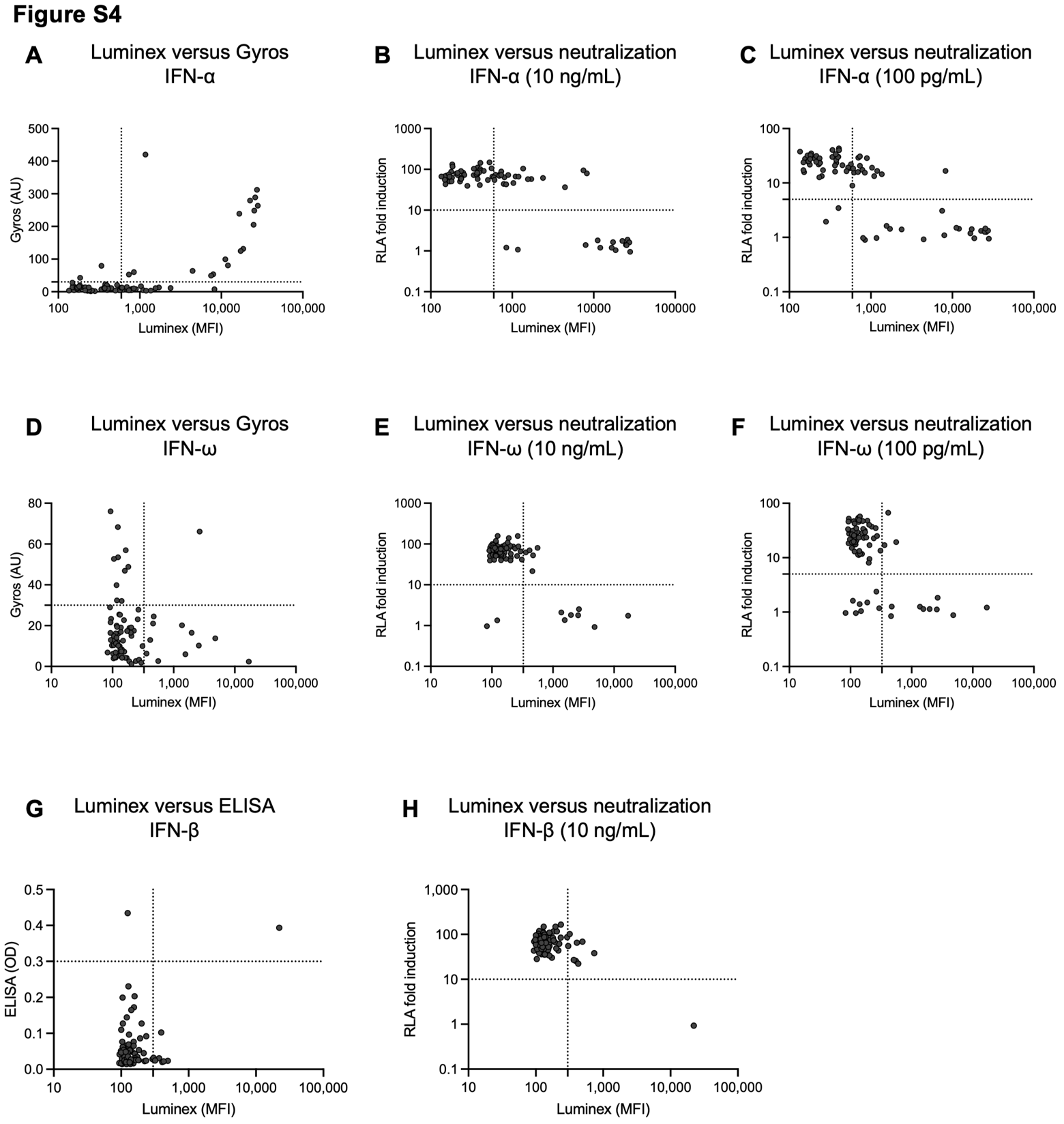


**Figure S4: Correlation of Luminex data with Gyros data and/or neutralization data for auto-Abs against IFN-α2, IFN-ω and IFN-β**.
